# Dissembled DJ-1 high molecular weight complex in cortex mitochondria from Parkinson's disease patients

**DOI:** 10.1186/1750-1326-4-23

**Published:** 2009-06-04

**Authors:** Hikmet Nural, Ping He, Thomas Beach, Lucia Sue, Weiming Xia, Yong Shen

**Affiliations:** 1 Haldeman Laboratory of Molecular and Cellular Neurobiology, Sun Health Research Institute, Sun City, Arizona, USA; 2 Civin Laboratory for Neuropathology and Brain Donation Program, Sun Health Research Institute, Sun City, Arizona, USA; 3 Center for Neurologic Diseases, Brigham and Women's Hospital, Boston, Massachusetts, USA

## Abstract

The PARK7 gene encodes a protein, DJ-1, with several functions such as protection of cells from oxidative stress, sperm maturation and fertilization, and chaperone activity. Mutations in the PARK7 gene are associated with autosomal recessive early-onset Parkinson's disease (PD). DJ-1 has been reported to be expressed in multiple cells in the central nerve system. Here, by using both native and denatured Western blots, we examined levels of total DJ-1 and high molecular weight complexes of DJ-1 (HMW) in both the substantia nigra and cortex from rapidly autopsied 18 PD and 9 non-pathological control (NPC) brains. We have discovered that the level of total DJ-1 protein is significantly reduced in the substantia nigra in brains of sporadic PD patients. Moreover, in the PD cortex mitochondria fraction, the HMW DJ-1 complex is significantly lower than in the NPC. These results suggest abnormal DJ-1 expression levels and DJ-1 complex changes may contribute to PD pathogenesis.

## Background

Parkinson's disease (PD), affecting 1% of the population over 65, is one of the most common neurodegenerative disorders characterized with selective loss of dopaminergic neurons in the substantia nigra [1-3]. Although molecular mechanism of disease is not fully known, studies indicate that mitochondrial dysfunction and oxidative stress could play a role in neuronal loss [4]. Three mutated genes – *Parkin, DJ-1*, and *PINK1 *– implicated in mitochondria and oxidative stress-related survival pathways are typically present in the brain with Parkinson's disease (PD) with apparent autosomal recessive inheritance. Two other genes – α *-Synuclein *and *LRRK2 *– are present in an autosomal dominant pattern and are associated with prominent intracellular protein inclusions [5,6]. A number of pathogenic mutations identified in the *DJ-1 *gene are linked to early onset familial Parkinson's disease (PD) [7-11]. Mechanistically, the DJ-1 protein has been reported to be sensitive to oxidative stress and it may function as an anti-oxidant protein [12-15].

DJ-1 is a small protein composing 189 amino acids and it is expressed in variety of tissues including brain [16,17]. Although its precise biochemical function is not fully known, recent studies indicate that it has been involved in many diverse biological processes including protease, chaperone and antioxidant activities [13,18-20]. Crystal structure and biochemical data show that DJ-1 forms dimers (37 KD) [18,20-23]. In addition to dimer formation, DJ-1 also tends to form high molecular weight (HMW) complexes (250–700 kD) during normal condition [21,24-26] It is possible that DJ-1 and other DJ-1 interacting proteins might form these HMW complexes. Although presence of α-synuclein is controversial [21,24,27], Parkin has been associated with DJ-1 HMW complex [21] However, whether the DJ-1 HMW complex remains intact in brains with sporadic PD is not clear. Regulation of the formation and distribution of the DJ-1 HMW complexes remains largely obscure at this stage. In this study, we analyzed the levels of DJ-1 HMW complex as well as the DJ-1 protein in the substantia nigra, temporal cortex and mitochondria fraction from the rapidly autopsied temporal cortex from the brains of PD patients by native and denatured Western blots. We found that the total DJ-1 protein was significantly reduced in the substantia nigra and the DJ-1 HMW complex in the cortex mitochondria is also significantly decreased in brains of sporadic PD patients. These results suggest abnormal DJ-1 expression levels and DJ-1 protein structure changes may contribute to PD pathogenesis.

## Materials and methods

### Patient brain tissue samples

Rapidly autopsied brain tissue was obtained from the Brain Donation Program, Sun Health Research Institute, Sun City, Arizona, USA. Human tissue was collected with informed consent from subjects or next of kin and with ethical approval from the Sun Health Institutional Review Board. Diagnosis of Parkinson's disease was made with the presence of Lewy bodies and pigmented neuron loss in the substantia nigra. Non-pathological controls (NPC) were selected based on the absence of Lewy bodies and/or neuron loss in the substantia nigra. Patients with autosomal recessive juvenile parkinsonism, a relatively rare syndrome that shares many of the features of parkinsonism but without the presence of Lewy bodies or Lewy neurites at autopsy [28,29], were excluded from this study. The average age of PD patients is 80 ± 6.9 years old and NPC is 85 ± 6.6 years old, therefore there were no significant difference in ages between PD and NPC (Table 1).

**Table 1:** General patient information and pathology criteria.

**Group**	**Sample Size (n)**	**Gender (M/F)**	**Age**	**Average Onset Age**	**Average Post-mortem**	**Pathological Summary**
PD	18	11/7	81 ± 6	67 ± 12	2.47 ± 0.72 hrs	Diagnosis of Parkinson's disease is made in the presence of a clinical diagnosis of Parkinsonism in which Lewy bodies and pigmented neuron loss are present in the substantia nigra.

NPC	9	5/4	84 ± 6		2.85 ± 0.48 hrs	Non-pathological controls (NPC) were selected based on absence of Lewy bodies and/or neuron loss in substantia nigra.

### Sample Preparation

Human brain tissues were preserved at -80°C until used. Grey matter from the temporal cortex was cut and re-suspended in re-suspension buffer (25 mM BisTris-HCl, 20% Glycerol, pH 7.0) plus proteinase inhibitors. One-tenth of the volume of the solubilization buffer (50 mM BisTris-HCl, 40% Glycerol, 2% SDS) was added, and followed by agitation at 4°C for 60 min. After centrifugation at 14000 rpm for 30 min, the supernatant was stored at -80°C.

Mitochondria purification was performed as described previously [30]. Briefly, brain tissue was homogenized in 0.32 M sucrose, 10 mM Tris-HCl, pH7.4, and spun at 900 g for 30 min. The supernatant was transferred to another tube and spun again at 900 g at 4°C. The mitochondria were then collected by centrifuging at 10,000 g for 30 min. The pellet was washed with 0.25 M sucrose three times and finally collected by centrifugation at 6500 g for 10 min. To examine the purity of the mitochondria, we used Western blotting to evaluate levels of mitochondria markers CoxIV (Abcam) and prohibitin (Abcam). To rule out a possibility of contamination with other cellular organs, we used EEA1 (BD Bioscience) as an endosome marker, GM130 (BD Bioscience) as a Golgi marker and LAMP2 (BD Bioscience) as a lysosome marker in purified lysates.

### Native and Denatured Gel electrophoresis and Western Blot

For native electrophoresis, samples were mixed with a 1:1 concentration of sample buffer (100 mM Tris-HCl pH6.8, 20%glycerol, 0.2% Bromophenol blue). Samples were separated on a 6% Tris-Glycine native gel (without SDS). For denatured electrophoresis, samples were diluted 1:1 with denaturing sample buffer (100 mM Tris-HCl pH6.8, 2.5%β-Mercapethanol, 4%SDS, 20%glycerol, 0.2%Bromophenol blue) and separated on a 10% standard SDS-PAGE, and then the protein was transferred to PDVF membrane. Western blots were probed by anti-DJ-1 polyclonal antibody DJ-1 raised against KLH-conjugated peptide NH2-KGAEEMETVIPVDVMRRAGCOOH corresponding to N-terminal residues 12–30 of human DJ-1 (1: 5,000) [21]. Blots were also probed with anti-β-actin monoclonal antibody (Sigma) as a loading control. For mitochondria samples, the blots were probed by an anti-CoxIV monoclonal antibody (1: 2,000) (Abcam) as a mitochondria loading control. Levels of high molecular weight DJ-1 complex and total DJ-1 were normalized to β-actin or CoxIV (for mitochondria samples).

### Statistical Analysis

Optical density of HMW and total DJ-1 in the substantia nigra, cortex and mitochondria from the cortex was analyzed and normalized to β-actin (substantia nigra, cortex) or CoxIV (mitochondria). One Way ANOVA was computed for the comparisons between PD and NPC groups. Total and HMW levels in PD are expressed as a percentage of (%) of NPC levels, and bars represent mean and ± SE.

### Primary cell culture and Immunofluorescence

Cells from the human superior temporal cortex were isolated and cultured according to our previous report [31]. The resulting cells were fixed with 4% paraformaldehyde for 10 min and Blocked with 10% goat serum for 30 min. Primary antibodies were applied with antibodies against β III tubulin (TUJ1, neuronal marker, Covance, 1:800), DJ1 (Chemicon, AB9212, 1:400), GFAP (astrocyte marker, Dako, 1:2,000), CoxIV (mitochondria marker, 1:1000). Cells were counterstained with DAPI (SantaCruz, 1:1,000). Fluorescent-labeling 488 (green) or 594 (red) secondary antibodies against rabbit IgG or mouse IgG were detected (Molecular Probes, 1:1000).

## Results

### Edged but significant difference in total DJ-1 levels in the Substantia Nigra between PD patients and NP controls

Since the major pathological symptom in sporadic PD is the presence of Lewy bodies in the substantia nigra, we first analyzed levels of DJ-1 in this region.

**Figure 1:**
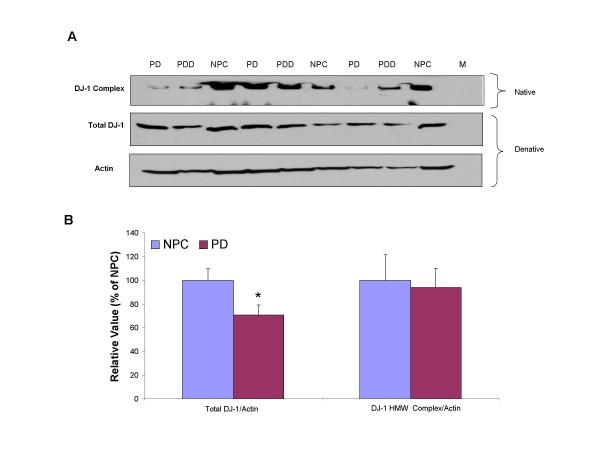
**Substantial Nigra DJ-1 levels in PD and NPC**. *A*. Substantia nigra samples were prepared and native protein was separated on a 6% Tris-glycine native gel and transferred to PDVF membrane. An equal amount of samples were mixed with denaturing sample buffer and separated on a 10% SDS-PAGE in parallel. Eighteen PD and 9 NPC were examined by Western blot. *B*. Spot density analysis of DJ-1 high molecular weight complex and total DJ-1 (denature gel) was normalized to β-actin level and indicated in percentage to NPC levels.

The DJ-1 HMW complex and total DJ-1 protein levels in the PD substantia nigra (n = 18) and age-matched NPC (n = 9) substantia nigra were compared by using native and denatured Western blotting (Fig. 1). The DJ-1 HMW complex was able to be detected in the substantia nigra tissue. After normalization to β-actin level, there was no significant difference in DJ-1 HMW complex between PD and NPC groups (*p *= 0.36) (Fig. 1B). However, though variation of total DJ-1 protein levels in PD and NPC were largely overlapped, we found that its level in PD is lower than NPC at an edge of significance (*p *= 0.047) (Fig. 1B). Quantitative analysis revealed that total DJ-1 reactivity was decreased by an average of 29% in PD compared to NPC, indicating that substantia nigra DJ-1 might be linked to PD progression.

### No significant change in DJ-1 levels in the Cortex of PD patients

**Figure 2:**
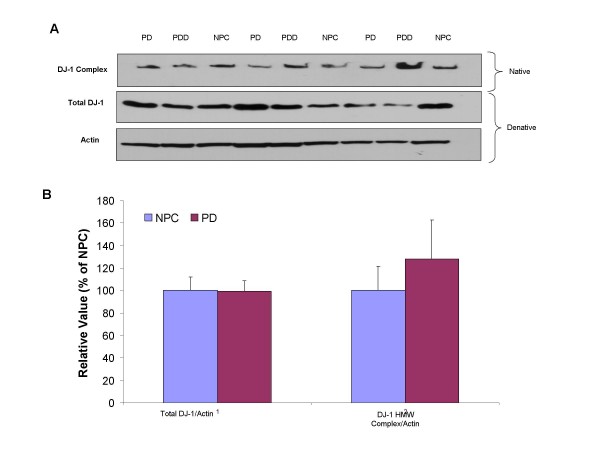
**STG DJ-1 levels in PD and NPC**. A. Super temporal cortex samples were prepared and analyzed by native and denature by Western blot. B. Spot density analysis of DJ-1 high molecular weight complex and total DJ-1 (denatured gel) was normalized to β-actin level and indicated in percentage to NPC levels.

Since oxidative stress was reported in the cortex region of sporadic PD patients [32], it would be interesting to find out if DJ-1 plays a role in regulating such stress in the cortex region. To examine whether HMW or total DJ-1 protein levels were affected in the cortex region of PD, protein levels of DJ-1 in the PD and NPC temporal cortex grey matter were examined by native and denatured Western blot (Fig. 2A). Quantitative band densitometry did not reveal any differences in the HMW complex (*p *= 0.22, Fig. 2B), or total DJ-1 (*p *= 0.42, Fig. 2B) levels between these two groups, indicating, as a whole, that DJ-1 protein levels in the cortex might not be related to PD phenotype.

### DJ-1 Is Localized in the Mitochondria of Both Glia and Neuron Cells

**Figure 3:**
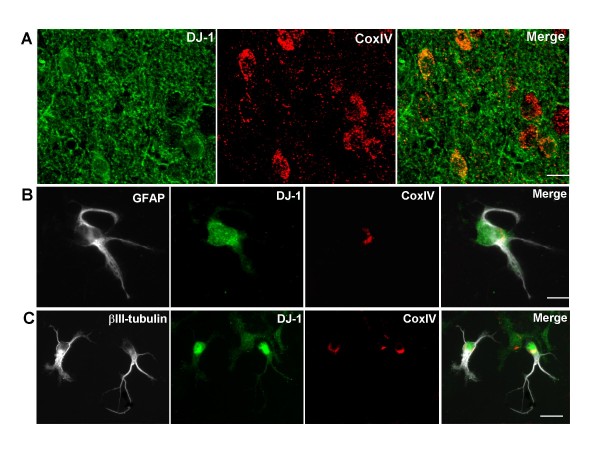
**DJ-1 is localized in mitochondria in both glial and neuronal cells**: A. Temporal cortex sections were double labeled with antibodies against DJ-1(green) and CoxIV (red). Co-localization of DJ-1 and CoxIV was showed in the merged picture. B. STG primary cell culture was stained with glial cell marker GFAP, followed by DJ-1 and CoxIV. Picture of DJ-1(green) and CoxIV (red) was merged to show co-localization of DJ-1 and mitochondria (yellow); C. STG primary cell culture was stained with neuron cell marker βIII-tubulin, followed by DJ-1 and CoxIV. Picture of DJ-1(green) and CoxIV (red) was merged to indicate co-localization of DJ-1 and mitochondria (yellow).

Studies have shown that DJ-1 is involved in protection against mitochondrial damage [14,15,33,34]. To find out whether DJ-1 exists in the mitochondria, we isolated primary cells (both neuron and astrocytes) from normal human brain cortex grey matter and immunostained with anti DJ-1 antibody and anti-CoxIV antibody (mitochondria marker) accompanied with glia cell marker, GFAP, or neuron cell marker, β III-tubulin. In glia cells (Fig. 3A), DJ-1 was widely distributed in the cytoplasm, while a portion of the DJ-1 protein co-localized with the mitochondria marker CoxIV (Fig. 3A), indicating that DJ-1is located in the glial cell mitochondria. Similarly, we also found that DJ-1 in neuronal cells is also localized to mitochondria (Fig. 3B). Our results suggested that, in both glia cells and neuron cells, a small portion of DJ-1 located in the mitochondria and could play a role in regulating oxidative stress in mitochondria, consistent with previous reports [26].

### HMW DJ-1 complex significantly decreased in PD cortex mitochondria

**Figure 4:**
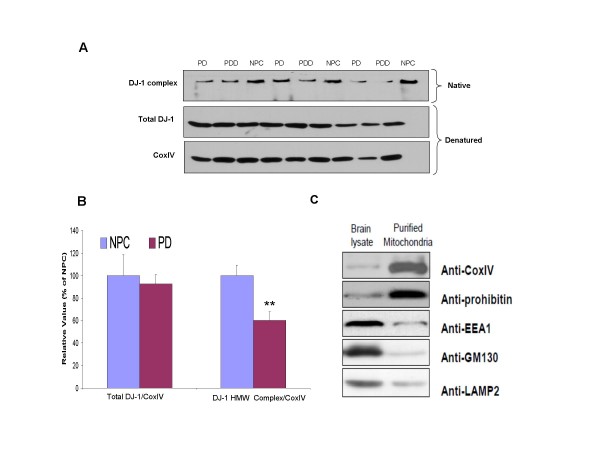
**STG grey matter mitochondria fraction DJ-1 levels in PD and NPC**: A. Super temporal cortex mitochondria were extracted and samples were prepared and analyzed by native and denatured Western blot. B. Spot density analysis of DJ-1 high molecular weight complex and total DJ-1 was normalized to CoxIV level and indicated in percentage to NPC levels. C. The Western blot study demonstrates cell compartments and related proteins in the mitochondria fraction. Specific antibodies that recognize specific cell compartments that are used, anti-CoxIV, anti-prohibitin are for mitochondria, anti-EEA1 is for early endosome antigen1 (EEA1), anti-GM130 is for Golgi Matrix protein (GM130) and anti-LAMP2 is for Lysosome-associated membrane protein-2 (LAMP2). When compare to the brain lysate before purification, CoxIV and prohibitin levels were found to be significantly elevated, more than 10 folds increased based on the densometric analysis, in the purified mitochondria. It shows that though there is little contamination of other cell organelles.

Native and denatured Western blotting was used to analyze the DJ-1 HMW complex, and total DJ-1 levels in the mitochondria fraction purified from the temporal cortex (Fig. 4A). After normalization to CoxIV, a mitochondria loading control, DJ-1 HMW complex levels were found to be significantly reduced by 39% in PD patients (*p = 0.005, Fig. 4B). No differences were observed in total DJ-1 levels (Fig. 4B). While total mitochondrial DJ-1 levels are unchanged; decrease in HMW complex levels indicates possible dissociation of DJ-1 or DJ-1 interacting protein from complex. Thus, indicating formation of HMW complex might play role in protection from oxidative stress in cortex of PD patients.

To examine the purity of the mitochondria isolated, we used Western blotting to evaluate levels of CoxIV and prohibitin, the mitochondria markers. When compared to the brain lysate before purification, CoxIV and prohibitin levels were found to be significantly enriched, more than a 10 fold increase based on the densometric analysis, in the purified mitochondria (Fig. 4C). This indicates that the purified mitochondria, by our purification method, are pure and enriched much more than non-purified brain lysates. However, there is still a possibility of traces of other organelles contaminating the results, which could be due to some overlap in the gradient centrifuge. To rule out such possibility, we examined the levels of other subcellular organelles by using EEA1, an endosome marker, GM130, a Golgi marker and LAMP2, a lysosome marker in purified lysates. We found that the cell organelle markers, including EEA1, GM130 and LAMP2 levels were significantly decreased (Fig. 4C), suggesting that although there could be other trace amounts of other organelles, the contribution of these contaminations were minimal.

Due to the large amount of tissue required to obtain a measurable amount of protein, which we were not able to obtain, substantia nigra mitochondrial fractions DJ-1 levels have not yet been examined.

## Discussion

*DJ-1 *is the third gene that has been linked to PD. Mutations in the *DJ-1 *gene cause early onset PD with autosomal recessive inheritance [7-11]. In this study, we have analyzed its levels in the more common sporadic form of PD. We analyzed HMW DJ-1 complex and total DJ-1 protein levels in PD patients. We found significantly lower total DJ-1 levels in the PD substantia nigra region, indicating reduction in DJ-1 protein levels might be related to the substantia nigra pathology in PD patients. In addition, we have shown that DJ-1 is localized in mitochondria of both neuronal and glial cells supporting the role of DJ-1 in mitochondrial function. When we compared the levels of total and HMW complex of DJ-1, we have discovered significantly decreased levels of HMW complex of DJ-1 in mitochondrial preparation of the cortex from PD brains.

We have found that total level of DJ-1 in the substantia nigra is decreased in PD compared to health controls (Fig. 1). However, other groups have reported either no change or increased levels of DJ-1 in PD brain [25,35-37]. One possibility of the discrepancy with previous groups is that they have not investigated DJ-1 specifically in the substantia nigra from PD brains. For instance, we could not observe any change in DJ-1 protein levels in the PD frontal cortex. Second, post-mortem interval (PMI) is shorter (< 2.5 hrs) and more consistent between PD and NPC in our study compared to other groups [25,35-37], which are critical factors. Third, we have analyzed a larger sample group (PD n = 18, NPC n = 9) compared to others. Variation and range within each group from smaller number of samples might affect the accuracy of the conclusion drawn from the data.

Oxidative stress has been well-documented in the substantia nigra in PD [12-15]. Recent studies have shown that over-expression of wild type DJ-1 in cultured human dopaminergic cells protected the cells from death induced by hydrogen peroxide and 6-hydroxydopamine, while over-expressing the L166P mutant DJ-1 had no such protective effect [34]; knocking down endogenous DJ-1 rendered cells susceptible to oxidative damage [34]. Also, wild type DJ-1 inhibited the mutant A53T α-synuclein-induced protein aggregation and cytotoxicity by increasing the expression of heat shock protein 70 [34]. Accordingly, our studies have shown that total levels of DJ-1 were significantly lower in the substantia nigra region of PD than that of NPC, implying that decreased DJ-1 levels might account for the loss of anti-oxidation capability in the substantia nigra region.

A recent study has also shown increased oxidative damage in the PD cortical region [32], but little has been reported on the subject. We proposed that DJ-1 HMW complex could account for this anti-oxidative stress function. However, since we did not observe any significant changes in the total DJ-1 levels in the cortex region, this suggests that the subcellular location of DJ-1, rather than the total amount of DJ-1, may be more important for its biological role. Recent studies in DJ-1 subcellular localization have identified an endogenous pool of DJ-1 in the mitochondrial matrix and inter-membrane space [26,38]. Therefore, DJ-1 is an integral mitochondrial protein that may have important functions in regulating mitochondrial physiology [26], consistent to our result that displayed co-localization of DJ-1 and mitochondria. Moreover, in response to oxidative stress, DJ-1 is translocated from cytosol to mitochondria and nucleus. DJ-1 tagged with mitochondrial signal confers more neuro-protection against oxidative stress when compared to WT or nuclear tagged DJ-1 in cell assays [39]. Thus, mitochondrial localization of DJ-1 is important to exert cell-protective effect in oxidative stress condition. Interestingly, we found that the DJ-1 HMW complex in the mitochondria fraction extracted from the PD cortical region is significantly lower than NPC. In DJ-1 HMW complex, Parkin, an E3-ubiquitin ligase, has been found [21]. Moreover, oxidative stress promotes association of Parkin and DJ-1[25]. Therefore, reduced levels of HMW complex in mitochondria isolated from PD cortex, may suggest reduced interaction or dissociation of DJ-1 with Parkin or possibly with other proteins as well. Additionally, recent studies also have shown that the subunit of mitochondrial complex I in the PD cortex region was oxidatively damaged and its function was impaired [40], implying that HMW DJ-1 complex might play a role in protecting the cortex mitochondria from oxidative stress.

Although total DJ-1 levels were reduced in substantia nigra, we could not observe any change in DJ-1 HMW complex levels in sustantia nigra. On the other hand, in cortex we could not observe any change in levels of DJ-1 HMW complex in the cortex of PD patients as well, but when we examined mitochondria enriched preparation from the cortex we observed a reduction in DJ-1 complex. It could be that DJ-1 levels or distribution of DJ-1 between HMW and dimers in mitochondria might be important for DJ-1 function. Therefore, it would be also interesting to examine the HMW and total DJ-1 levels in the substantia nigra mitochondria. However, due to the large amount of tissue required to prepare the mitochondria fraction, while relatively small amount of the substantia nigra tissue was available, mitochondrial fractions from substantia nigra has not yet been examined until we collect enough tissue for this study in the future.

It is worthwhile to note that large variations were seen with each group, despite the fact that all samples were taken from the same brain region. It is practically impossible to obtain a sample set from patients in the same cohort with identical genetic backgrounds, which would reduce the variation. In order to clearly distinguish PD and NPC, we categorized our patients based on pathological definitions. The inherent heterogeneity of the samples meant that we could not rule out other genetic factors that could affect DJ-1 levels. Future studies are anticipated to analyze DJ-1 HMW complex and total DJ-1 in the mitochondria fraction of substantia nigra, when a sufficient amount of brain tissue is available for biochemical analysis, and to investigate the possible mechanisms involved.

## Competing interests

The authors declare that they have no competing interests.

## Authors' contributions

HN analyzed the results and performed the statistical analysis, and revised the manuscript PH participated in acquisition of data and performed immunohistochemistry and immunocytochemistry experiments. Both HN and PH did trouble shooting to ensure experimental results reliable. TB and LS involved in collection of tissue used in this study. WX and YS designed experiments. YS supervised HN and PH in experiments and participated in preparation of the manuscript. All authors read and approved the final manuscript.
