# Disassembled DJ-1 high molecular weight complex in cortex mitochondria from Parkinson's disease patients

**DOI:** 10.1186/1750-1326-4-30

**Published:** 2009-07-15

**Authors:** Zhenyu Zhong, Hikmet Nural, Ping He, Gina Civarella, Thomas Beach, Lucia Sue, Charles Adler, Holly Shill, John Caviness, Weiming Xia, Yong Shen

**Affiliations:** 1 Haldeman Laboratory of Molecular and Cellular Neurobiology, Sun Health Research Institute, Sun City, AZ USA; 2 Civin Laboratory for Neuropathology and Brain and Body Donation Program, Sun Health Research Institute, Sun City, AZ USA; 3 Parkinson's Disease and Movement Disorders Center, Mayo Clinic, Department of Neurology, Scottsdale, AZ USA; 4 Cleo Roberts Center for Clinical Research, Sun Health Research Institute, Sun City, AZ USA; 5 Center for Neurologic Diseases, Brigham and Women's Hospital, Boston, MA USA; 6 Neuroinflammatory Lab, Sun Health Research Institute, Sun City, AZ USA

## Abstract

Correction to Nural H, He P, Beach T, Sue L, Xia W, Shen Y. Disassembled DJ-1 high molecular weight complex in cortex mitochondria from Parkinson's disease patients *Molecular Neurodegeneration *2009, 4:23.

## Correction

After publication of this work [1], we noted that we inadvertently failed to include the complete list of all coauthors. The full list of authors has now been added and the Authors' contributions and competing interests section modified accordingly.

## Competing interests

The authors declare that they have no competing interests.

## Authors' contributions

ZZ prepared samples, performed western blot experiments and statistic analysis of the data as well as wrote manuscript. HN involved in analyzing the results and the statistical analysis, and revised the manuscript; PH participated in acquisition of data and performed immunohistochemistry and immunocytochemistry experiments. ZZ, HN and PH did trouble shooting to ensure experimental results reliable. GC helped revising the manuscript. TB and LS involved in collection of tissue used in this study; CA, HS and JC helped clarifying patients' clinical information. WX and YS designed experiments. YS supervised HN and PH in experiments and participated in preparation of the manuscript. All authors read and approved the final manuscript.
